# Beyond supplements and surrogates: an evidence-quality map informs a strategic rebalancing of dietary research in prostate cancer active surveillance

**DOI:** 10.3389/fnut.2026.1911038

**Published:** 2026-08-10

**Authors:** Ying Li, Tian-Qi Wang, Ai-Yun Zhu, Yin-Ping Zhang, Qiang Fu

**Affiliations:** 1 Department of Urology, Shandong Provincial Hospital Affiliated to Shandong First Medical University, Jinan, Shandong, China; 2 Department of Urology, Shandong Provincial Hospital, Jinan, Shandong, China; 3 School of Nursing, Health Science Center, Xi’an Jiaotong University, Xi’an, Shaanxi, China

**Keywords:** prostate cancer, active surveillance, dietary patterns, evidence gaps, bias

## Abstract

**Background:**

Evidence regarding whether dietary interventions can delay disease progression in men undergoing active surveillance for prostate cancer remains fragmented. This study innovatively combines a systematic review of the evidence with rigorous methodological assessment, aiming to provide a critical analysis of the evidence base in this field, rather than merely a descriptive review.

**Methods:**

We searched the PubMed, Embase, Cochrane Library, and Web of Science databases, mapping interventions to outcomes. An evidence bubble plot was constructed, with the bubble size representing the number of studies and the color intensity representing the proportion of randomized controlled trials. Methodological quality were assessed using the Cochrane risk of bias tool, ROBINS-I, the Newcastle–Ottawa Scale and the JBI tool.

**Results:**

The map revealed a dense but flawed evidence cluster focused on phytochemical-rich supplements and PSA/biomarker change, primarily supported by randomized controlled trials with prevalent limitations in blinding and randomization reporting. In stark contrast, there is a critical gap in the evidence from intervention studies assessing the impact of holistic dietary patterns on clinical progression endpoints. The integrated quality assessment underscores a fundamental mismatch: while abundant, research on supplements is often methodologically compromised, conversely, the few observational studies on holistic dietary patterns, although of higher quality, are unable to establish causality.

**Conclusion:**

Current evidence is misaligned with clinical needs, being rich in low-certainty supplement data but scarce in high-quality trials on holistic diets. Future research would benefit greatly from a strategic reorientation of funding—namely, prioritizing large-scale, rigorous clinical trials to evaluate comprehensive dietary interventions targeting clinically significant disease progression endpoints, while recognizing that supplement studies continue to play an important role in generating mechanistic hypotheses.

**Systematic review registration:**

https://doi.org/10.17605/OSF.IO/T8G97

## 1 Introduction

Prostate cancer (PCa) is the most common malignancy among men worldwide (1). For patients with low-risk and select cases of intermediate-risk localized PCa, active surveillance (AS) has gradually become the preferred management strategy over immediate curative treatment (2, 3), with the goal of balancing tumor control with quality of life while avoiding treatment-related complications. However, studies have shown that among PCa patients treated with AS, approximately 20%–50% will eventually experience disease progression and require curative treatment (4). During the lengthy period of AS, patients with PCa face considerable uncertainty regarding their condition and often experience severe psychological stress (5). Consequently, identifying effective, safe, and feasible non-pharmacological interventions that could delay disease progression and lengthen the surveillance interval is highly valuable.

Dietary intervention, as a modifiable lifestyle factor that is low-cost and offers potential synergistic benefits such as anti-inflammatory and antioxidant effects, is among the most common non-pharmacological interventions and has attracted considerable attention in the field of cancer prevention (6). Nevertheless, the specific role of dietary intervention in men who are already diagnosed with PCa and who are managed with AS remains unclear (6, 7). The current evidence remains fragmented: relevant studies range from analyses of individual nutrients to holistic dietary patterns, with findings spanning everything from intermediate biomarkers to clinical decision-making, and have often yielded inconsistent conclusions. Consequently, clinicians lack reliable, evidence-based guidance and are unable to answer the key question posed by patients: among the many types of dietary interventions available, what exactly should PCa patients on AS eat to effectively help control disease progression?

An evidence map is a method for synthesizing evidence. By systematically collecting, evaluating and synthesizing existing evidence, it clarifies the current state of research and identifies gaps, thereby facilitating in-depth research and decision-making (8). Traditional systematic reviews and meta-analyses are suited for evaluating the efficacy of mature, specific interventions. In this field, where research is still exploratory, the primary need is to clarify “what evidence exists,” “how it is connected,” and “what is missing.” Therefore, this study is the first to employ evidence map, integrated with a critical appraisal of methodological quality.

This approach extends the descriptive scope of traditional reviews and aims to: provide a clear overview of the distribution and intensity of evidence linking dietary interventions to disease outcomes, critically assess the reliability of existing evidence, particularly in the most heavily researched areas, and accurately identify gaps in high-quality evidence, rather than merely noting the absence of studies, thereby providing an evidence-based rationale for reorienting research investment toward issues of greatest clinical significance to patients, while acknowledging the valid but limited role of mechanism-based research focused on dietary supplements.

Furthermore, we recognize that biomarkers are indispensable for establishing biological plausibility, providing evidence of mechanisms of action, and calculating sample sizes for future trials. However, the key bottleneck lies not in biomarker research itself, but in the persistent inability to translate promising biomarker signals into clinical endpoint trials with sufficient statistical power. Our evidence map is also designed to shed light on this translational gap.

## 2 Materials and methods

### 2.1 Study design and reporting

This work was a systematic evidence map study, reported according to best-practice guidelines for evidence synthesis to ensure transparency and reproducibility (8) (Supplementary File 1). Early in the study, a stakeholder advisory group comprising urologists, PCa patients and nutritionists was established to ensure the clinical relevance of the research questions and the classification framework. Patient representatives explicitly prioritized two outcomes—delaying treatment escalation and reducing biopsy-related anxiety—over mere changes in biomarkers, which directly led us to decide to focus on clinical progression endpoints in the outcome categories of the evidence map. Other patient-centered outcomes, including conversion to active treatment, MRI progression, quality of life, and adherence, were also discussed during stakeholder meetings; however, due to their sparse reporting across the included studies, they could not serve as primary classification categories in the evidence map. Study protocol registered on Open Science Framework^1^.

### 2.2 Evidence sources and search strategy

A systematic search was conducted in the PubMed, Embase, Cochrane Library, and Web of Science Core Collection databases from January 2000 to December 2025, the search was limited to English-language publications. The search strategy was developed based on the PICO (Population: men with PCa on AS; Intervention: any dietary intervention; Comparator: usual diet, no intervention, or placebo; Outcome: disease progression, biomarker changes, quality of life, etc.) framework. The full strategy is provided in Supplementary File 2.

### 2.3 Study selection and data extraction

All the retrieved records were imported into EndNote X9 for deduplication. Two independent reviewers screened titles/abstracts and subsequently full texts against predefined eligibility criteria. Discrepancies were resolved by consensus or with a third reviewer.

Inclusion criteria: Studies involving adult men with histologically confirmed PCa on AS; investigations of any dietary intake or nutritional supplements; and studies including randomized controlled trials (RCTs), cohort, case–control, and analytical cross-sectional studies. The exclusion criteria were studies involving patients receiving active treatment or with metastatic disease, those not reporting AS-specific outcomes, non-English publications, or studies for which the full text was unavailable.

Data on study characteristics, participants, interventions, comparators, outcomes, and key findings were extracted using a standardized form (Supplementary File 3) (7, 9–29).

### 2.4 Evidence map

Classification and Mapping: Based on extracted data and team discussion, interventions were categorized into six classes: holistic dietary patterns, other types, omega-3/fish oil, vitamin D3, phytochemical-rich supplement (PRS), selenium/folic acid. Classification combined data-driven clustering (based on reported interventions) and concept-driven categorisation (distinguishing holistic patterns from single supplements by biological rationale). The “other types” category includes high-vegetable and calorie-restricted diets. Outcomes were grouped into three classes—PSA, non-PSA biomarkers, and Gleason grade—to capture the spectrum from surrogate markers to the primary clinical endpoint determining treatment escalation. PSA was treated separately as the most standardized AS monitoring tool. Non-PSA biomarkers include tissue-based (e.g., Ki-67), hormonal, and gene expression markers. Gleason grade was selected as the sole clinical progression indicator because it is the primary determinant of treatment escalation in AS protocols and the most consistently reported hard endpoint. Each study was assigned to an intervention–outcome intersection according to its primary intervention and primary endpoint (Supplementary File 3).

Quantification: The total number of studies and the number of RCTs within each intersection were calculated.

**FIGURE 1:**
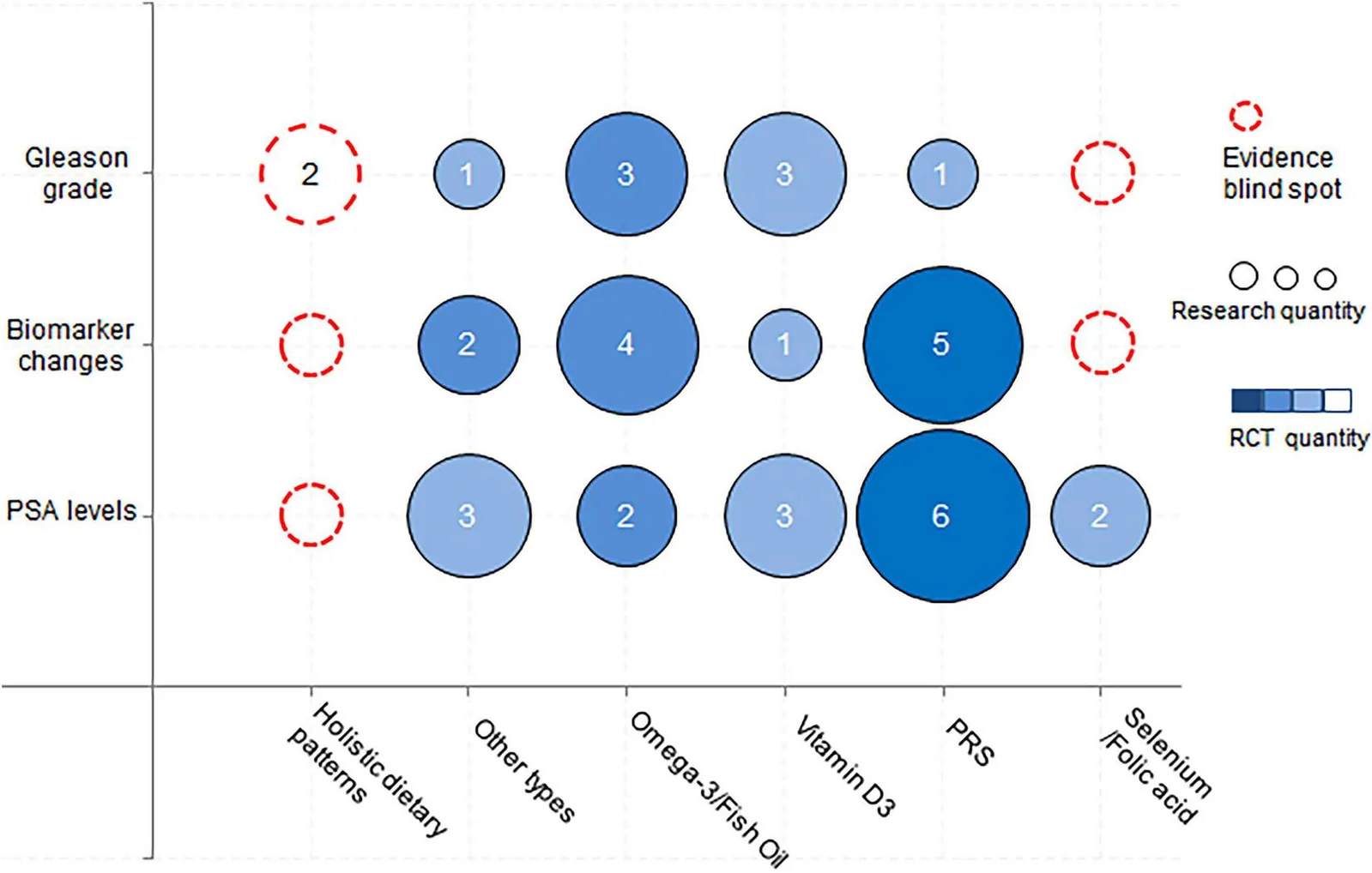
Evidence bubble plot. Evidence bubble plot showing distribution of 22 studies by intervention and outcome category. Bubble size indicates number of studies, color intensity indicates proportion of randomized controlled trials (RCTs). Red dashed circles mark evidence gaps.

Visualization: An evidence bubble plot was generated (Figure 1). The X-axis represents the intervention categories, and the Y-axis represents the outcome categories. The position of a bubble denotes a specific “intervention–outcome” pair. Bubble size is proportional to the total number of studies. The shade of the bubble color (light to dark blue) represents the proportion of RCTs, with darker blue indicating a greater concentration of RCTs. Intersections with zero studies are explicitly marked with a red dashed circle, defined as an “evidence blind spot.”

### 2.5 Assessment of methodological quality and risk of bias

Two independent reviewers performed the assessments using design-specific tools: The Cochrane Risk of Bias tool for RCTs (30); ROBINS-I for non-randomized interventional studies (31); cohort studies were assessed using the Newcastle-Ottawa Scale (NOS) (32); and cross-sectional studies were assessed using the JBI critical appraisal checklist (33).

## 3 Results

### 3.1 Study selection and characteristics

**FIGURE 2:**
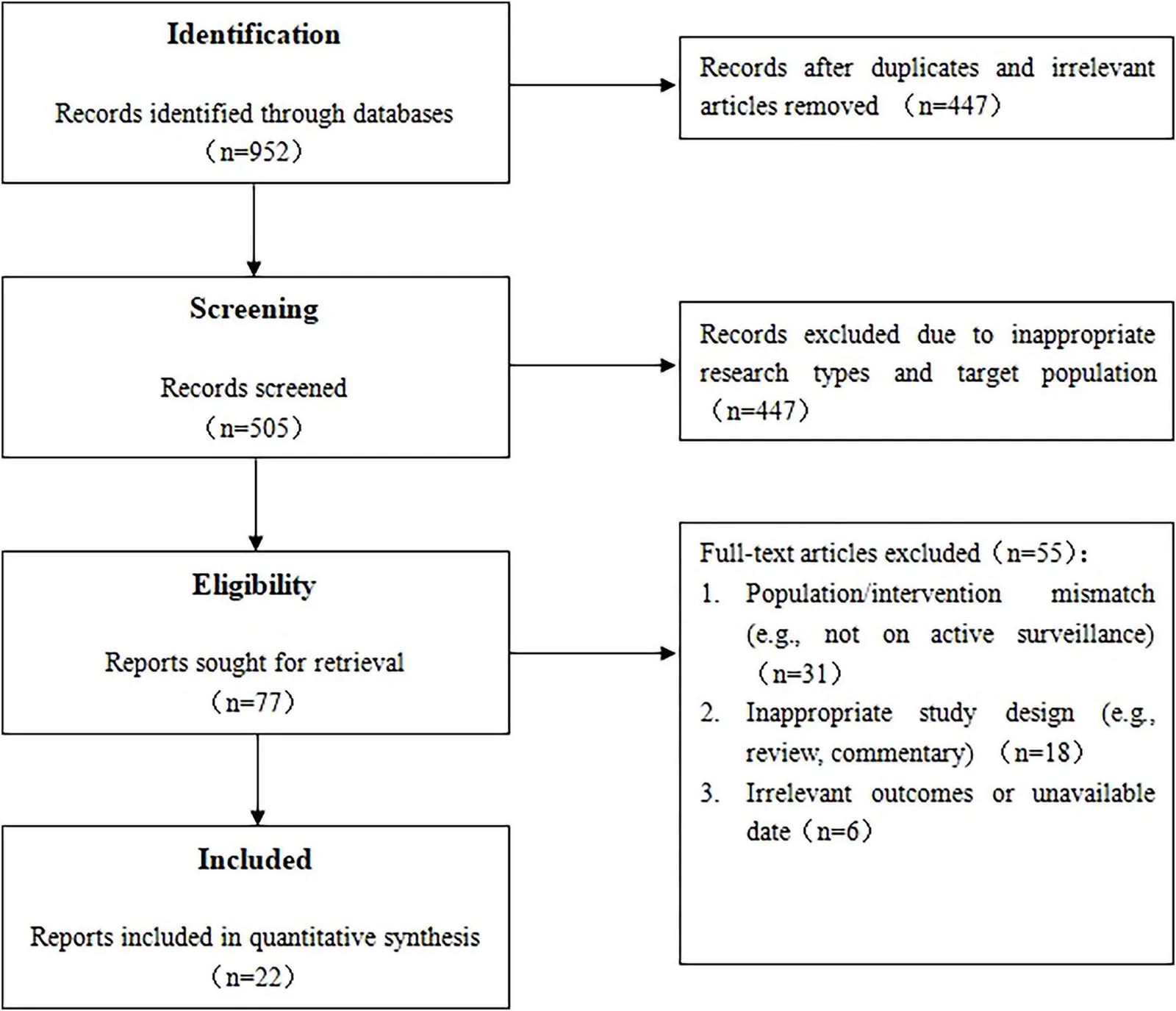
PRISMA flowchart.

The initial search yielded 952 records. After deduplication and screening, 22 studies were included: 12 RCTs, six non-randomized interventional studies, and four observational studies (two cohort studies and two cross-sectional studies). The study selection process is detailed in the PRISMA flowchart (Figure 2).

### 3.2 Evidence map and quadrant matrix

The bubble plot (Figure 1) and quadrant matrix (Table 1) reveals three key features: High-density, moderate-strength evidence clusters: The intersection of “PRS–Biomarker changes” and “PRS–PSA levels” forms the largest and darkest bubble, indicating that this is the most active area of research, driven primarily by RCTs. Secondary clusters existed for “Omega-3/fish oil–Biomarkers/Gleason grade.”

**TABLE 1:** Evidence quadrant matrix: from descriptive mapping to actionable priorities.

Research density Methodological robustness	High methodological robustness	Low methodological robustness
High research density	Consolidation zone (Ready for synthesis) empty	Re-evaluation zone (Methodological recalibration needed) PRS→biomarkers/PSA
Low research density	Prioritization zone (Priority for RCTs) holistic diet→gleason grade	Evidence deserts zone (Foundational exploration needed) holistic diet→PSA/biomarkers

Blind spots: The most prominent blank areas were the intersections of “holistic dietary patterns” with “PSA levels” and “biomarker changes” (marked by red dashed circles). Notably, while the intersection of “holistic dietary patterns” and “Gleason grade” contained two prospective cohort studies, indicating exploratory associations, the complete absence of interventional studies (especially RCTs) renders it a “blind spot” from the perspective of high-quality evidence needed to verify causality. Evidence linking “composite dietary interventions” to clinical hard endpoints was also extremely weak.

To move beyond descriptive visualization, we classified each evidence cluster into four actionable quadrants based on research density and methodological robustness (Table 1). The PRS–PSA/Biomarker cluster fell into the “Re-evaluation Zone,” whereas the holistic diet–Gleason cluster occupied the “Prioritization Zone.” The “Consolidation Zone” was empty across all intersections. The holistic diet–PSA/Biomarkers fell into the “Evidence Deserts Zone.”

### 3.3 Results of methodological quality and risk of bias assessment

**FIGURE 3:**
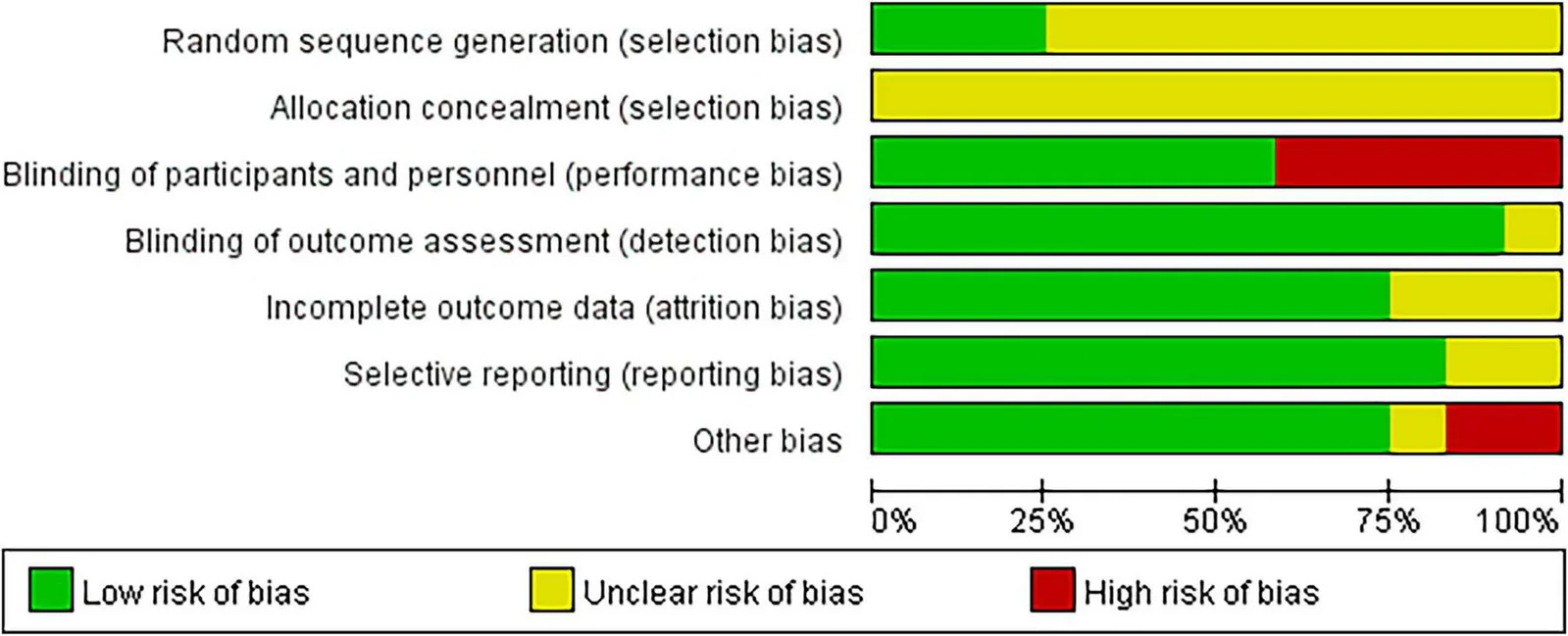
Randomized controlled trial (RCT) risk of bias graph.

**FIGURE 4:**
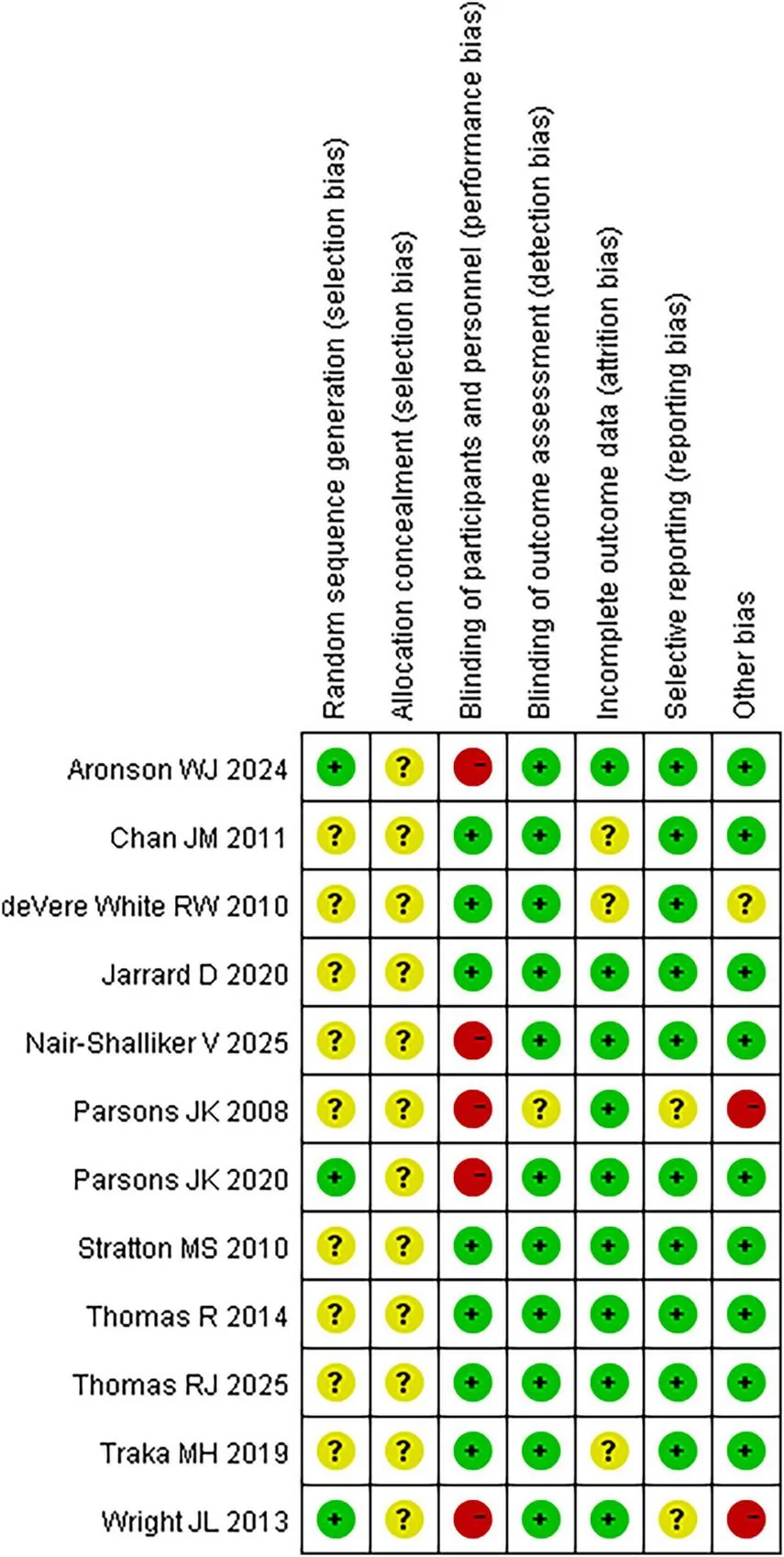
Randomized controlled trial (RCT) risk of bias summary.

Randomized controlled trials: Using the Cochrane risk-of-bias tool, we assessed all 12 RCTs (Figures 3, 4 and Supplementary File 4). The overall risk was judged as “high” in 2 studies (17%) and “moderate” in 10 studies (83%). At the domain level: random sequence generation was rated “unclear” in nine studies (75%); allocation concealment was “unclear” in all 12 studies (100%); blinding of participants and personnel was “high risk” in five studies (42%); blinding of outcome assessment was “unclear” in two studies (17%); incomplete outcome data was “unclear” in three studies (25%); selective reporting was “unclear” in two studies (17%); and other bias was “high” in one study (8%) and “unclear” in one study (8%). No study was rated high risk for outcome measurement, incomplete outcome data, or selective reporting. Cross-validation using the updated 2.0 framework confirmed this pattern: 75% of studies received “some concerns” predominantly due to unclear randomization reporting, while the minority of “high risk” judgments were confined to the blinding domain.

**FIGURE 5:**
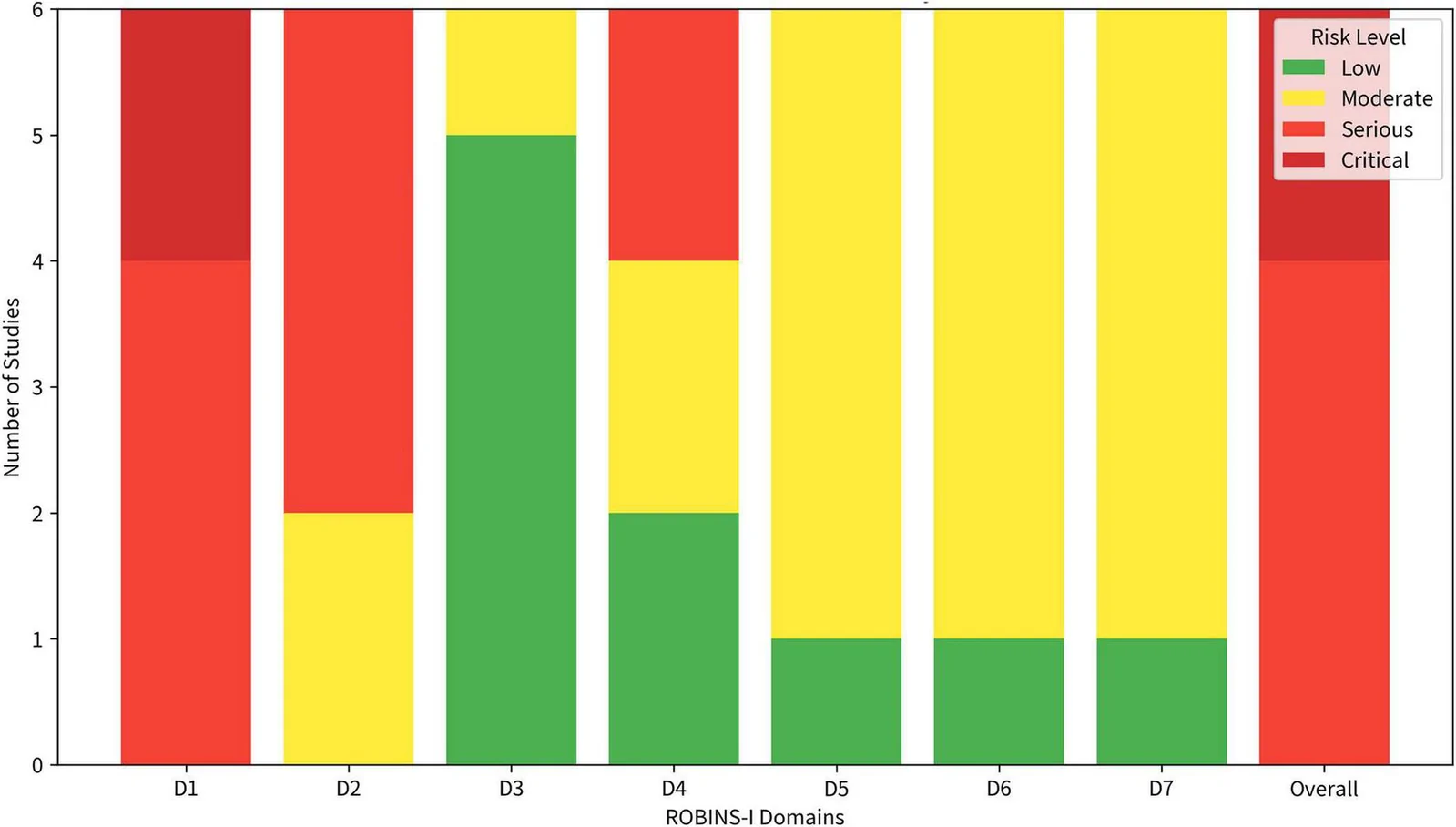
ROBINS-I risk of bias summary. D1, random sequence generation; D2, allocation concealment; D3, blinding of participants and personnel; D4, blinding of outcome assessment; D5, incomplete outcome data; D6, selective reporting; D7, other bias.

A cautious interpretation of non-randomized studies: This study assessed six non-randomized intervention studies using the ROBINS-I tool (Figure 5 and Supplementary File 4). The overall risk of bias in these studies was rated as “high” or “serious,” which severely undermines the reliability of the evidence. The most critical issues were centered on “confounding bias” and “participant selection bias.”

Strengths and limitations of observational studies: Both cohort studies received high scores (8 stars and 7 stars, respectively) following assessment using the NOS, indicating good methodological quality despite some limitations regarding exposure confirmation and the adequacy of follow-up (Supplementary File 3). The two analytical cross-sectional studies were assessed using the JBI Critical Appraisal Checklist and were rated as “high quality.” They clearly defined the study population, employed reliable measurement methods, and attempted to control for major confounding factors.

### 3.4 Description of key evidence clusters

Holistic dietary patterns: Only one high-quality cohort studies with not entirely consistent conclusions, suggesting potential benefit. This dietary pattern may reduce the risk of Gleason score progression, but it offers protective effects against chronic diseases and mortality. The absence of RCT evidence represents the primary translational chasm (7, 9).

Omega-3 fatty acids/fish oil: One recent RCT of high quality suggested a reduction in prostate tissue Ki-67 index, but the ROB 2.0 assessment indicated “some concerns” regarding randomization and blinding. Cross-sectional studies hinted that tissue biomarkers might be more predictive (10–13).

PRS: This cluster had the highest volume of research, with multiple RCTs reporting positive effects on biomarkers or PSA. However, quality appraisal indicated that these positive-finding RCTs were predominantly open-label or inadequately blinded, carrying risks of performance and detection bias. Furthermore, the translation of these biological effects to clinical progression remains unconfirmed (14–21).

Vitamin D3: Early non-randomized studies showed promising signals (22, 23). However, the most recent RCT failed to demonstrate efficacy in preventing disease progression (24), highlighting the need for high-quality RCTs.

## 4 Discussion

### 4.1 Diagnosis: what the evidence map reveals

#### 4.1.1 Overview of principal findings

The evidence map reveals a dense but flawed cluster of evidence focused on PRS and their effects on biomarkers (including PSA), primarily based on RCTs. however, these trials generally have limitations regarding blinding and randomization reporting. In stark contrast, there is a significant gap in the evidence regarding intervention studies evaluating the impact of holistic dietary patterns on clinical progression endpoints. A comprehensive quality assessment highlights a fundamental mismatch: while there is a large volume of research on dietary supplements, it is often plagued by methodological flaws; conversely, the few observational studies on holistic dietary patterns, although of higher quality, are unable to establish causality.

Given the significant heterogeneity among the included studies in terms of intervention type, definition of the control group, follow-up duration, and outcome reporting, conducting a formal meta-analysis would be inappropriate and unlikely to yield meaningful pooled estimates. Therefore, we adopted a structured narrative review, combining the visual findings from the evidence map with a systematic quality assessment.

#### 4.1.2 Analytical insights from the quadrant matrix

Our evidence map based on quadrant-based classification, yields three analytical insights that would be obscured by simply counting the number of studies. First, the “Re-evaluation Zone” indicating that the number of studies in this area is negatively correlated with methodological reliability, this paradox suggests that accumulating more such studies would yield little benefit. Second, the holistic evidence cluster covering diet and Gleason grade is located in the “Prioritization Zone,” indicating that while the signals from observational studies are sufficient to justify investment in RCTs, they are not yet sufficient to guide clinical recommendations. Third, the “Consolidation Zone”—where all intersection points are blank—constitutes a key finding in itself: no research area within this field has yet reached the threshold for a synthesis of conclusive evidence, highlighting that the entire research endeavor remains in its early stages.

#### 4.1.3 Redefining evidence gaps: the “Sandwich” situation

Traditional gap analysis focuses on areas of “research gap.” By integrating multiple research designs and quality assessment methods, our evidence map proposes a more critical definition: a lack of high-quality interventional evidence. The overall association between diet and the Gleason score is a prime example—two high-quality cohort studies provide evidence of an association, but the lack of RCT evidence directly hinders the translation of epidemiological findings into clinical practice.

Neither observational studies nor non-randomized trials have been able to bridge this gap: while cohort studies are methodologically rigorous, they are limited to hypothesis generation; non-randomized trials, on the other hand, suffer from severe confounding bias. Consequently, the current evidence base presents an alarming “sandwich” scenario: the top layer (interventional studies using clinical endpoints) is missing; the middle layer (RCTs) has significant methodological flaws; and the bottom layer (observational studies) cannot establish causality. Positive signals are more common in small, exploratory studies using surrogate endpoints, whereas larger, multicenter studies have consistently failed to demonstrate differences in biopsy-confirmed disease progression—a finding that reflects methodological heterogeneity rather than a lack of evidence supporting dietary interventions themselves.

### 4.2 Analysis: why the misalignment exists

#### 4.2.1 Structural factors contributing to research imbalance

Why does this disconnect between study scale and clinical relevance exist? Several factors may contribute to this situation. In terms of feasibility, RCTs evaluating standardized supplements offer research “convenience,” as they are easy to conduct with blinding, placebo controls, and the ability to obtain surrogate endpoint data in a short period. However, long-term trials testing the impact of complex dietary patterns on disease clinical progression face multiple severe challenges, including long follow-up periods, poor patient compliance, high costs, and complex confounding control (34). With respect to endpoint selection, although biomarkers are indispensable for exploring mechanisms, excessive focus on them in this field has created a self-reinforcing evidence chain that does not effectively connect to clinical outcomes that genuinely concern patients. During active monitoring, the predictive validity of many biomarkers for long-term clinical outcomes in PCa patients has not yet been verified. Additionally, compared with research aimed at public health dietary recommendations, commercial funding may be more inclined to support supplemental studies that can be patented and commercialized (35), potentially resulting in a mismatch between the generated evidence and the broad health benefits for patients.

#### 4.2.2 Biomarkers in the translational pipeline: reframing the issue

A balanced interpretation of our findings requires explicit acknowledgment that biomarkers are not the problem—they are an essential part of the solution. Their roles are well-established: establishing biological plausibility, providing early proof-of-mechanism, enabling sample size calculations for definitive trials, and identifying subpopulations most likely to benefit. The issue our map reveals is not that biomarker studies exist, but that the translational pipeline from biomarker signal to clinical endpoint validation remains consistently uncompleted in this field.

To address this, future research should adopt an integrated, phase-aligned approach: Phase I/II studies should continue using biomarkers for mechanism discovery and signal detection, with rigorous methodological standards (blinding, objective measurement, pre-registration). Phase III trials should then prioritize clinical progression endpoints (e.g., time to treatment escalation, Gleason grade reclassification), while embedding biobanking and biomarker substudies within these larger trials to simultaneously test efficacy and refine mechanistic understanding. This design—clinical efficacy and biomarker discovery pursued in parallel within the same trial—would transform the current “either/or” tension into a “both/and” synergy, making holistic dietary trials scientifically richer rather than less rigorous.

### 4.3 Prescription: a strategic roadmap forward

#### 4.3.1 Filling the blind spots: differentiated strategies for different zones

The quadrant matrix not only diagnoses this problem but also directs its solution. For the “Evidence Deserts Zone” (Holistic diet–PSA/Biomarkers), the immediate priority is foundational descriptive studies—prospective cohorts with serial biomarker measurements to establish temporal trajectories and effect size estimates, which are prerequisites for power calculations in future trials. For the “Prioritization Zone” (holistic diet–Gleason progression), where two high-quality cohort studies already provide consistent signals, the logical next step is a pragmatic cluster-randomized trial embedded within existing AS registries, using a simplified dietary pattern intervention with clinical progression as the primary endpoint and biomarker adherence checks as secondary validation. Notably, the selenium/folate cluster also falls within the “Evidence Deserts Zone” by our criteria, but the existing two studies—both with unclear randomization/blinding and null or even harmful signals—suggest this gap may be better deprioritized. These targeted strategies ensure that research investment matches the maturity level of each evidence gap, guided not merely by convenience or feasibility, but by the potential to generate high-quality evidence that addresses the outcomes patients value most.

#### 4.3.2 Making holistic diet trials feasible: addressing adherence, cost, confounding, and follow-up duration

To translate these findings into a viable research agenda, future holistic dietary trials should adopt a pragmatic, phased design that addresses issues such as adherence, cost, and confounding factors. Regarding adherence, we recommend combining smartphone-based real-time dietary recording with validation using objective biomarkers (such as plasma carotenoids and urinary polyphenols) and simplified dietary pattern scores (such as the Modified Mediterranean Diet Score) to reduce the burden on participants while ensuring verifiable adherence. To control costs, practical trials embedded within existing clinical cohort infrastructure can leverage established recruitment and follow-up systems and adopt a phased funding approach—beginning with an initial feasibility pilot, starting with a small-scale, single-center, 6-month feasibility trial to establish compliance benchmarks and effect size estimates. Only after meeting predefined feasibility thresholds should the study proceed to larger-scale confirmatory trials, such as multicenter, long-term (≥5 years) practical RCTs. Regarding control of confounding factors, we advocate for cluster randomization by study site to minimize dietary contamination, the use of multiple imputation or inverse probability weighting to handle missing data, and careful stratification based on baseline dietary habits and key clinical covariates. From feasibility studies and dose determination, through preliminary efficacy trials, to confirmatory Phase III trials, this stepwise framework acknowledges the inherent complexity of dietary intervention research while providing a practical and resource-efficient implementation pathway.

#### 4.3.3 Why rebalance toward holistic dietary patterns? A four-dimensional rationale

Why should future investment rebalance toward holistic dietary patterns rather than continuing primarily with supplementation trials? The rationale rests on four converging lines of evidence.

First, biological plausibility: The onset and progression of prostate cancer are driven by multiple interacting signaling pathways. Changes in dietary patterns may reduce systemic inflammation, improve signaling along the insulin-insulin-like growth factor (IGF) axis, modulate lipid mediators, and influence the tumor microenvironment—effects that cannot be replicated by a single supplement targeting just one signaling pathway (6, 36). To translate these effects into clinically meaningful outcomes (such as delaying the need to escalate treatment intensity), sufficient intervention “dose,” objective verification of adherence, and a longer follow-up period than in most existing trials are required. Second, epidemiological evidence: Large-scale cohort studies consistently show that the Mediterranean diet or the DASH diet are associated with reduced all-cause mortality and cancer-specific mortality (36), whereas a small number of dietary supplement trials have repeatedly shown no effect or even harmful effects, for example, high-dose selenium supplementation is a risk factor for an increased rate of PSA rise, suggesting that the effects of “whole diets” exceed the sum of the effects of their individual components. Third, feasibility: Patients consume food, not dietary supplements; recommending sustainable dietary patterns is more scalable than complex daily supplement regimens. Fourth, cost and safety: The marginal cost of adopting a healthier diet is low, whereas proprietary dietary supplements are expensive and carry a higher risk of toxicity.

These four considerations suggest that supplement research remains valuable for proposing mechanistic hypotheses, but resource allocation should gradually shift back toward holistic dietary trials—not because supplement research is without value, but because the evidence from biology, epidemiology, practicality, and safety mutually reinforces the notion that a holistic approach represents a more promising investment for improving patient-related outcomes (37).

#### 4.3.4 Enhancing methodological rigor: distinguishing design constraints from reporting deficits

Interpreting the ROB findings requires distinguishing between two qualitatively different issues. The “unclear” judgments—predominantly in random sequence generation (75%) and allocation concealment (100%)—reflect incomplete reporting, not methodological failure. In contrast, the “high risk” judgments (42% for blinding of participants and personnel) reflect a genuine design constraint inherent to dietary trials: maintaining blinding over extended periods is inherently difficult. However, its impact on validity depends on outcome objectivity—centrally adjudicated endpoints are less vulnerable. The risk of bias in non-randomized studies remains consistently high, limiting their contribution to hypothesis generation rather than definitive conclusions.

These two issues point toward different remedies. Reporting gaps can be addressed through adherence to CONSORT guidelines. The blinding challenge can be mitigated by prioritizing objective, centrally-adjudicated primary endpoints (e.g., pathological upgrading) and employing blinded outcome assessors. Discussion sections should candidly acknowledge open-label limitations while clarifying which aspects of validity remain robust. Developing a field-specific consensus on methodological best practices for dietary intervention trials in AS populations would help standardize quality expectations and facilitate meaningful evidence synthesis.

#### 4.3.5 Integrating patient perspectives: what matters most to men on AS

First, patients experience periodic anxiety about disease progression, which motivates them to actively seek dietary adjustments—yet they feel deeply uncertain about which adjustments are truly beneficial (38). Second, compared to improvements in biomarkers alone, patients place greater value on treatment outcomes that maintain quality of life and minimize invasive procedures, which confirms our emphasis on clinical progression endpoints. Third, the barriers to sustained dietary adjustments—including a lack of clear guidance and challenges with actual adherence—highlight the need to prioritize feasibility in future trial designs. These insights ensure that trial design and the selection of outcome measures are grounded in patients” primary concerns, while not superseding the necessity of conducting rigorous RCTs.

### 4.4 Strengths and limitations

The main strength of this study lies in its innovative combination of an evidence map with a systematic assessment of methodological quality, providing a multidimensional and critical overview of the field. The visual representation of the map powerfully highlights structural imbalances and mismatches in the research, thereby redefining the concept of “evidence gaps.” The main limitation lies in the inherent subjectivity of the classification frameworks for interventions and outcomes. Furthermore, the small number of studies within some clusters restricts more in-depth subgroup analysis.

## 5 Conclusion

This study reveals a significant resource imbalance in dietary research for PCa on AS: substantial investment has been directed toward supplements and surrogate endpoints with methodological limitations and uncertain clinical relevance, while high-quality evidence on holistic dietary patterns and long-term clinical outcomes is lacking. Most supplements lack sufficient evidence to delay progression, and general healthy dietary recommendations remain the cornerstone of clinical practice. Our evidence gap map can guide personalized nutritional plans for AS patients, aiming to improve quality of life and delay progression. We call on the research community, funding agencies, and journals to support a strategic rebalancing of research investment—prioritizing outcomes of greatest clinical value to patients, as defined by patients themselves, while maintaining a constructive role for supplement research in mechanistic hypothesis generation.

## Data Availability

The original contributions presented in this study are included in this article/Supplementary material, further inquiries can be directed to the corresponding authors.

## Footnotes

1
https://doi.org/10.17605/OSF.IO/T8G97
