# Supplementary material for: Beyond supplements and surrogates: an evidence-quality map informs a strategic rebalancing of dietary research in prostate cancer active surveillance
Source: Front Nutr. 2026 Aug 10;13:1911038. doi: 10.3389/fnut.2026.1911038 (PMC13500317; doi:10.3389/fnut.2026.1911038)
Supplement: Supplementary file 1 [file Table_1.doc]

Section	Topic	Item #	Checklist
Title	Title	1	Specify the scope and identify the report as a mapping review, evidence and gap map, or both
Authors	Authors	2	List names and affiliations of all authors
Abstract	Structured summary	3	Provide a structured summary that includes (as applicable) background, including the rationale and objective for the review*; methods, including stakeholder engagement, protocol, eligibility criteria, search, coding, critical appraisal, and data presentation and analyses (specifically mention the strategy for adequacy and priority setting); results, including study selection, characteristics, risk of bias, and mapping analyses; conclusions, including a summary of the main findings and implications of those findings
Background	Rationale	4	Describe the scope of the review and explain why conducting this review is important
	Objectives	5	Provide a structured statement of the research question(s) within a key element framework, and specify whether this review is intended for decision-making, to delineate evidence gaps and clusters for future research, or both. Utilize frameworks such as PI/ECOS (Population, Intervention/Exposure, Comparator, Outcome, and Study Design), PCC (Population, Concept, and Context), PIT (Population, Index Test, Target Condition), and others like PECO, PEO, and PO for various research questions.
Methods	Stakeholders		
	Identification and definition of stakeholders	6a	Specify the identification process of stakeholders and define the various types of stakeholders involved, such as direct users of research outputs (e.g., researchers and policy decision-makers), and those directly affected by decisions (e.g., patients in the field of medicine)
	Stakeholder engagement	6b	Provide detailed descriptions of stakeholder engagement at each stage of the review process
	Registration and protocol		
	Registration information	7a	Provide registration information for the review, or state if the review was not registered
	Reference protocol	7b	Apply the review protocol, or specify if no protocol was established
	Deviations from protocol	7c	Describe and explain any amendments made to the information provided at registration or in the protocol
	Eligibility criteria	8	Specify the inclusion and exclusion criteria for the review, defining characteristics of the study within a key element framework based on different research questions.
	Search sources	9	Present all databases, registers, websites, organizations, reference lists, and other sources searched or consulted to identify studies, along with the dates when each source was last searched or consulted. If machine learning was utilized for literature search, present the details of the sources from which the literature was drawn
	Search strategy	10	Present the full search strategies for all search sources, including any filters and limits used. If machine learning was utilized for literature search, present the details of the relevant software and its search strings
	Screening process	11	Provide the process for literature screening, including at the title/abstract and full texts levels, and clarify the methods ensuring the repeatability of this process, such as specifying the number of people involved and whether they worked independently. If machine learning was used for literature screening, specify the details of how inclusion decisions were made
	Data extraction and coding		
	Data items	12a	List all variables used for data extraction and/or further coding
	Development of coding tools	12b	Define and provide details on the analytic or consensus-based conceptual framework developed for developing coding tools
	Data collection process	13	Provide the process for data extraction from reports of included studies and coding, and clarify the methods ensuring the repeatability of this process, such as specifying the number of people involved and whether they worked independently. If machine learning was used for data extraction, specify the details of the relevant software and the logic used for extracting key fields.
	Critical appraisal	14	Specify whether the quality of included studies or reviews was assessed. If done, describe the methods used for assessment and the procedures ensuring the repeatability of the assessment process, such as specifying the number of people involved and whether they worked independently.
	Data presentation and analysis		
	Types of presentation	15a	Specify the presentation formats for the mapping results of included studies, such as charts, tables, and interactive maps
	Tools for mapping	15b	Describe the tools or software details used for generating mapping results, including any automation or artificial intelligence software
	Dimensions in Maps	15c	Specify the dimensions or coordinate matrix and filters (if possible) used for positioning within the maps. If Evidence Gap Maps (EGMs) tools are utilized, consult the PRISMA-EGM guidelines as necessary
	Strategy for adequacy and priority setting	15d	Specify the strategy for determining areas with sufficient evidence, aimed at supporting decision-making and future research priorities, including evidence gaps and clusters. If possible, specify the method for ranking these priorities.
	Data analysis methods	15e	Describe the methods used in the data analysis process, such as descriptive analysis, thematic analysis, and statistical analysis, among others
Results	Study selection		
	Flow of studies	16a	Describe the results of the search and selection process, from the number of references initially identified to the number of studies ultimately included in the review. Provide a flow diagram to illustrate this process
	Excluded studies	16b	List key excluded studies that readers might reasonably expect to find and provide justification for each exclusion
	Study characteristics	17	Describe the basic study characteristics of interest. Consider equity and provide the citations, native format of extracted data and its corresponding coding for each included study, if possible.
	Quality assessments	18	If quality was assessed, describe the quality assessments for included studies or reviews.
	Mapping analysis		
	Maps of included studies	19a	Present a map here, showing how the relevant literature is organized according to transparent, replicable key elements framework (research question), with a concise description. If possible, consider equity and include filters that allow for the generation of customized maps.
	Areas with adequate evidence	19b	Provide a structured report within a key element framework detailing areas with sufficient evidence support for decision-making based on the strategy for adequacy, if possible
	Evidence gaps and clusters	19c	Provide a structured report within a key element framework detailing areas requiring further research, including primary studies and additional evidence synthesis, based on the strategy for priority setting, if possible
Discussion	Summary of main results	20	Describe the main findings and provide a general interpretation of the results within the context of other evidence
	Limitations of the review	21	Discuss the limitations of both the review processes and the included evidence
	Implications	22	Discuss the implications for decision-making and future research in a structured manner based on key elements framework, if possible
	Plans for map updates	23	Discuss the necessity of updating the map based on current research results and trends, and provide details of the update plan, including timing and content to be updated.
Conclusions	Conclusions	24	Report the main findings of the review and summarize the implications of those findings
Acknowledgements	Acknowledgements	25	Acknowledge the contributions of individuals who are not listed as authors of the review
Contributions of authors	Contributions of authors	26	Detail the specific contributions of each author
Declarations of interest	Declarations of interest	27	Report any competing interests of the review authors
Sources of support	Sources of support	28	Describe the sources of financial or non-financial support for the review and specify the role
