# Supplementary material for: Beyond supplements and surrogates: an evidence-quality map informs a strategic rebalancing of dietary research in prostate cancer active surveillance
Source: Front Nutr. 2026 Aug 10;13:1911038. doi: 10.3389/fnut.2026.1911038 (PMC13500317; doi:10.3389/fnut.2026.1911038)
Supplement: Supplementary file 2 [file Table_2.doc]

**The search strategy**

**PubMed** **:** **number 185**

("Prostatic Neoplasms"[Mesh] OR "Prostatic Neoplasm"[Title/Abstract] OR "Prostate Cancer"[Title/Abstract] OR "Prostate Cancers"[Title/Abstract]) AND ("Watchful Waiting"[Mesh] OR "watchful waiting"[Title/Abstract] OR "active surveillance"[Title/Abstract] OR "expectant management"[Title/Abstract] OR "conservative management"[Title/Abstract] OR "deferred therapy"[Title/Abstract] OR "deferred treatment"[Title/Abstract]) AND (Diet[Mesh] OR diet[Title/Abstract] OR dietary[Title/Abstract] OR nutrition*[Title/Abstract] OR "Nutritional Sciences"[Mesh] OR "Nutritional Status"[Mesh] OR Food[Mesh] OR food[Title/Abstract] OR "Diet Therapy"[Mesh] OR "Dietary Supplements"[Mesh] OR supplement*[Title/Abstract] OR "Micronutrients"[Mesh] OR "Diet, Mediterranean"[Mesh] OR "Mediterranean diet"[Title/Abstract] OR "Diet, Vegetarian"[Mesh] OR "vegetarian diet"[Title/Abstract] OR "Diet, Plant-Based"[Mesh] OR "plant-based diet"[Title/Abstract] OR "dietary pattern*"[Title/Abstract] OR "Lycopene"[Mesh] OR lycopene[Title/Abstract] OR "Tomatoes"[Title/Abstract] OR "Vitamin D"[Mesh] OR "Vitamin D"[Title/Abstract] OR "Vitamin E"[Mesh] OR "Vitamin E"[Title/Abstract] OR "Fish Oils"[Mesh] OR "Fatty Acids, Omega-3"[Mesh] OR "omega-3"[Title/Abstract] OR "Selenium"[Mesh] OR selenium[Title/Abstract] OR "Brassica"[Mesh] OR cruciferous[Title/Abstract] OR broccoli[Title/Abstract] OR "Soy Foods"[Mesh] OR soy[Title/Abstract])

**Embase: number 439**

('prostate cancer'/exp OR 'prostatic neoplasm'/exp OR 'prostate cancer':ti,ab OR 'prostatic neoplasm':ti,ab) AND ('watchful waiting'/exp OR 'active surveillance'/exp OR 'watchful waiting':ti,ab OR 'active surveillance':ti,ab OR 'expectant management':ti,ab OR 'conservative management':ti,ab OR 'deferred therapy':ti,ab OR 'deferred treatment':ti,ab) AND ('diet'/exp OR 'nutrition'/exp OR 'food'/exp OR 'diet therapy'/exp OR 'diet supplementation'/exp OR 'micronutrient'/exp OR 'mediterranean diet'/exp OR 'vegetarian diet'/exp OR 'plant based diet'/exp OR 'lycopene'/exp OR 'tomato'/exp OR 'vitamin D'/exp OR 'vitamin E'/exp OR 'fish oil'/exp OR 'omega 3 fatty acid'/exp OR 'selenium'/exp OR 'brassica'/exp OR 'soybean'/exp OR 'soy food'/exp OR diet:ti,ab OR dietary:ti,ab OR nutrition*:ti,ab OR food:ti,ab OR supplement*:ti,ab OR 'mediterranean diet':ti,ab OR 'vegetarian diet':ti,ab OR 'plant-based diet':ti,ab OR 'dietary pattern*':ti,ab OR lycopene:ti,ab OR tomato*:ti,ab OR 'vitamin d':ti,ab OR 'vitamin e':ti,ab OR 'omega-3':ti,ab OR selenium:ti,ab OR cruciferous:ti,ab OR broccoli:ti,ab OR soy:ti,ab OR soybean*:ti,ab)

**Web of Science Core Collection: number 208**

TS=(("prostatic neoplasms" OR "prostatic neoplasm" OR "prostate cancer" OR "prostate cancers") AND ("watchful waiting" OR "active surveillance" OR "expectant management" OR "conservative management" OR "deferred therapy" OR "deferred treatment") AND (diet OR dietary OR nutrition* OR food OR supplement* OR "diet therapy" OR "dietary supplements" OR "micronutrients" OR "mediterranean diet" OR "vegetarian diet" OR "plant-based diet" OR "dietary patterns" OR lycopene OR tomato* OR "vitamin d" OR "vitamin e" OR "fish oils" OR "omega-3 fatty acids" OR "omega-3" OR selenium OR cruciferous OR broccoli OR soy OR soybean* OR "soy foods" OR "nutritional sciences" OR "nutritional status"))

**Cochrane Library: number 145** **Trials 120**

#1	(prostate near cancer) OR (prostatic near neoplasm) OR "prostate cancer" OR "prostatic neoplasm"	19761

#2	("watchful waiting") OR ("active surveillance") OR ("expectant management") OR ("conservative management") OR ("deferred therapy") OR ("deferred treatment")	5477

#3	(diet OR dietary OR nutrition* OR food OR supplement*) OR ("diet therapy") OR ("dietary supplements") OR ("micronutrients") OR ("mediterranean diet") OR ("vegetarian diet") OR (plant NEXT based NEXT diet) OR (dietary NEXT pattern*) OR (lycopene) OR (tomato*) OR ("vitamin d") OR ("vitamin e") OR ("fish oils") OR ("omega-3") OR (selenium) OR (cruciferous) OR (broccoli) OR (soy OR soybean*) OR ("nutritional sciences") OR ("nutritional status")	278836

#4	#1 AND #2 AND #3	145 Trials 120
