# Supplementary material for: Beyond supplements and surrogates: an evidence-quality map informs a strategic rebalancing of dietary research in prostate cancer active surveillance
Source: Front Nutr. 2026 Aug 10;13:1911038. doi: 10.3389/fnut.2026.1911038 (PMC13500317; doi:10.3389/fnut.2026.1911038)
Supplement: Supplementary file 3 [file Table_3.doc]

**Table 1 Data extraction form**

| **Author (Year)** | **Country** | **Research**  **Design** | **Sample size** | **Diet categories and follow-up time** | **Intervention**  **me****asures** | **Control group** | **Outcomes and key findings** |
| --- | --- | --- | --- | --- | --- | --- | --- |
| Schenk JM,2023 | USA | Cohort study | 565 | Holistic dietary pattern  3 years | High adherence to dietary patterns assessed by FFQ: HEI-2015, aMED, or DASH. | None | Higher adherence to HEI-2015, aMED, or DASH dietary patterns was not significantly associated with a reduced risk of grade reclassification, and have well-established protective effects on chronic  diseases and mortality. |
| Gregg JR,2019 | USA | Cohort study | 411 | Holistic dietary patterns  a median follow-up of 36 months | High diet quality at baseline, defined as the highest of the Healthy Eating Index (HEI)-2015 score. | None | Gleason grade progression.  higher diet quality or conformance with United States dietary guidelines at enrolment may lower risk of Gleason grade progression, though additional confirmatory research is needed. |
| Parsons JK,2008 | USA | RCT | 43 | Other types  6 months | Telephone-based dietary (seven servings per day of vegetables) | Printed material with standard guidelines recommending five servings of vegetables and fruits daily | Telephone-based dietary counselling increases vegetable intake and plasma concentrations of potentially anticarcinogenic carotenoids in men with prostate cancer on active surveillance. |
| Parsons JK,2020 | USA | RCT | 478 | Other types  2 years | Telephone promoting consumption of 7 or more daily vegetable servings | Receive written information about diet | Not significantly reduce the risk of prostate cancer progression. |

**Table 1 (Continued)**

| **Author (Year)** | **Country** | **Research**  **Design** | **Sample size** | **Diet categories and follow-up time** | **Intervention measures** | **Control group** | **Outcomes and key findings** |
| --- | --- | --- | --- | --- | --- | --- | --- |
| Wright JL,2013 | USA | RCT | 19 | Other types  6 weeks | Caloric-Restricted diet | Continue their current diet | A significant difference from baseline in weight loss, dietary constituent intake, and IGFBP-3 levels in the intervention group compared to the control group. |
| Aronson WJ,2025 | USA | RCT | 100 | Omega-3 / fish oil  1 year | High omega-3, low omega-6 fatty acid diet with fishoil capsules (D+FO) | No dietary intervention | A significant reduction in Ki-67 index. |
| Moreel X,2014 | Canada | Cross-sectional study | 48 | Omega-3 fatty acids | Omega-3 fatty acids | None | Prostate tissue w-3 fatty acids, especially EPA, may be protective against prostate cancer progression in men with low-risk prostate cancer. |
| Moussa H,2019 | USA | Cross-sectional study | 157 | Omega-3 | Omega-3 fatty acids | None | LCω3-EPA levels in the target prostate tissue are inversely associated with high-grade PCa, supporting that prostate tissue FA, but not RBC FA, is a reliable biomarker of PCa risk. |
| Ullevig SL,2021 | USA | Non-randomized Studies | 28 | Folic acid  12 weeks | Folic acid reduction diet. | None | Reduced dietary folic acid intake from food and supplements and successfully lowered serum and RBC folate without resulting harmful effects. |

**Table 1 (Continued)**

| **Author (Year)** | **Country** | **Research**  **Design** | **Sample size** | **Diet categories and follow-up time** | **Intervention measures** | **Control group** | **Outcomes and key findings** |
| --- | --- | --- | --- | --- | --- | --- | --- |
| Nair-Shalliker V,2025 | Australia | RCT | 123 | Vitamin D  24 months, | Vitamin D supplementation | Placebo | Vitamin D supplementation did not prevent PC progression, although reduced prevalence of CBMN markers may indicate a benefit of vitamin D supplementation. |
| Hollis BW,2013 | USA | Non-randomized Studies | 44 | Vitamin D  1 year | Vitamin D3 (4000IU/d) | Baseline and the historical control group | Robust and sustained vitamin D3 supplementation can reduce prostate cancer-related health disparities in African-American men and that these health disparities are at least in part the result of widespread hypovitaminosis D within the African-American population. |
| Marshall DT,2012 | USA | Non-randomized Studies | 52 | Vitamin D3  1 year | Vitamin D3 soft gels (4000 IU) | Baseline | No significant changes in PSA levels were observed. A decrease in the number of positive cores or decrease in Gleason score. |
| Jarrard D,2020 | USA | RCT | 30 | PRS  1 year | Pomegranate fruit extract (PFE) 1000mg | Placebo | PFE contains bioactive compounds capable of altering biomarkers involving oxidative stress and androgen signaling in prostate tumor and normal‐appearing adjacent tissue. No alterations in the IGF axis were noted. |

**Table 1 (Continued)**

| **Author (Year)** | **Country** | **Research**  **Design** | **Sample size** | **Diet categories and follow-up time** | **Intervention**  **measures** | **Control group** | **Outcomes and key findings** |
| --- | --- | --- | --- | --- | --- | --- | --- |
| Traka MH,2019 | UK | RCT | 49 | PRS  1 year | From 1 of 2 experimental broccoli genotypes with enhanced concentrations of glucoraphanin, delivering 3 and 7 times that of the control | A weekly 300 mL portion of soup made from a standard broccoli | Inversely correlate diets rich in either cruciferous vegetables or glucosinolates with prostate cancer incidence or progression. |
| Thomas RJ,2025 | UK | RCT | 208 | PRS  4 months | PRS + probiotic capsule | Placebo | Influence PCa progression and improve their symptoms. |
| Thomas R,2014 | UK | RCT | 199 | PRS  6 months | An oral capsule containing a blend of pomegranate, green tea, broccoli, and turmeric | Placebo | A significant short-term, favourable effect on the percentage rise in PSA. |
| Van der Eecken H,2024 | Belgium | Non-randomized Studies | 150 | PRS  1 year | Isoflavones (10mg) and fermented soy (equol) (60mg), 1capsule daily | None | A fermented soy supplement may be useful in helping select patients who can remain under AS or who need to switch to active therapy. |
| deVere White RW,2004 | USA | Non-randomized Studies | 62 | PRS  6 months | Capsules containing the genistein-rich extract three times daily by mouth. | None | As a sole treatment did not reduce PSA levels by 50% or morein 51 of 52 subjects and did not appear to be an effective treatment when given alone. |

**Table 1 (Continued)**

| **Author (Year)** | **Country** | **Research**  **Design** | **Sample size** | **Diet categories and follow-up time** | **Intervention measures** | **Control group** | | **Outcomes and key findings** | |  |
| --- | --- | --- | --- | --- | --- | --- | --- | --- | --- | --- |
| deVere White RW, 2010 | USA | RCT | 53 | PRS  6 months | A supplement containing 450 mg genistein, 300 mg daidzein, and other isoflavones daily | | Placebo | | Although high amounts of aglycone isoflavones may result in significantly elevated serum concentrations of genistein and daidzein, these dietary supplements alone did not lower PSA levels in men with low-volume prostate cancer. | |
| Naiki T, 2025 | Japan | Non-randomized Studies | 5 | PRS  6 months | luteolin, a flavonoid daily with 50 mg of oral luteolin | | None | | AEs were not noted in any patients for six months.  serum micro(mi)RNA expression in serum and prostate gland, including miR-29 and miR-30, tended to be upregulated after luteolin treatment. | |
| Chan JM, 2011 | USA | RCT | 69 | Folic acid  3 months | Lycopene, 3 g/day fish oil (including 1,098 mg eicosapentaenoic and 549 mg docosahexaenoic fatty acids) | | Placebo | | Not significantly change IGF-1 and COX-2 gene expression in the normal prostate micro-environment. | |
| Stratton MS, 2010 | USA | RCT | 140 | Selenium  5 years | Selenium p.o. 200 or 800 microg/d | | Placebo | | Selenium supplementation did not show a protective effect on PSA velocity in subjects with localized prostate cancer. On the contrary, supplementation with high-dose selenium was observed to be a risk factor for increased PSA velocity in men with high baseline plasma selenium concentrations. | |
