# Supplementary material for: Beyond supplements and surrogates: an evidence-quality map informs a strategic rebalancing of dietary research in prostate cancer active surveillance
Source: Front Nutr. 2026 Aug 10;13:1911038. doi: 10.3389/fnut.2026.1911038 (PMC13500317; doi:10.3389/fnut.2026.1911038)
Supplement: Supplementary file 4 [file Table_4.doc]

The Cochrane risk-of-bias assessment tool for randomised trials (RCT-ROB )

| Domain | Random sequence generation | Allocation concealment | Blinding of participants and personnel | Blinding of outcome assessment | Incomplete outcome data | Selective reporting | Other bias | Overall bias |
| --- | --- | --- | --- | --- | --- | --- | --- | --- |
| Parsons JK, 2008 | Unclear | Unclear | High | Unclear | Low | Unclear | High | High |
| Parsons JK, 2020 | Low | Unclear | High | Low | Low | Low | Low | Moderate |
| Wright JL, 2013 | Low | Unclear | High | Low | Low | Unclear | High | High |
| Aronson WJ, 2024 | Low | Unclear | High | Low | Low | Low | Low | Moderate |
| Jarrard D, 2020 | Unclear | Unclear | Low | Low | Low | Low | Low | Moderate |
| Traka MH, 2019 | Unclear | Unclear | Low | Low | Unclear | Low | Low | Moderate |
| Thomas RJ, 2025 | Unclear | Unclear | Low | Low | Low | Low | Low | Moderate |
| Thomas R, 2014 | Unclear | Unclear | Low | Low | Low | Low | Low | Moderate |
| deVere White RW, 2010 | Unclear | Unclear | Low | Low | Unclear | Low | Unclear | Moderate |
| Chan JM, 2011 | Unclear | Unclear | Low | Low | Unclear | Low | Low | Moderate |
| Stratton MS, 2010 | Unclear | Unclear | Low | Low | Low | Low | Low | Moderate |
| Nair-Shalliker V, 2025 | Unclear | Unclear | High | Low | Low | Low | Low | Moderate |

Version 2 of the Cochrane risk-of-bias assessment tool for randomised trials (RCT-ROB 2.0)

| Bias domain and signalling question | Bias arising from the randomisation process | Bias due to deviations from intended interventions | Bias due to missing outcome data | Bias in measurement of the outcome | Bias in selection of the reported result | Overall bias |
| --- | --- | --- | --- | --- | --- | --- |
| Parsons JK, 2008 | Some Concerns | High | Low | Some Concerns | Some Concerns | High |
| Parsons JK, 2020 | Some Concerns | High | Low | Low | Low | High |
| Wright JL, 2013 | Low | High | Low | Low | Some Concerns | High |
| Aronson WJ, 2024 | Low | Some Concerns | Low | Low | Low | Some Concerns |
| Jarrard D, 2020 | Some Concerns | Low | Low | Low | Low | Some Concerns |
| Traka MH, 2019 | Some Concerns | Low | Some Concerns | Low | Low | Some Concerns |
| Thomas RJ, 2025 | Some Concerns | Low | Low | Low | Low | Some Concerns |
| Thomas R, 2014 | Some Concerns | Low | Low | Low | Low | Some Concerns |
| deVere White RW, 2010 | Some Concerns | Low | Some Concerns | Low | Low | Some Concerns |
| Chan JM, 2011 | Some Concerns | Low | Some Concerns | Low | Low | Some Concerns |
| Stratton MS, 2010 | Some Concerns | Low | Low | Low | Low | Some Concerns |
| Nair-Shalliker V,2025 | Low | Some Concerns | Low | Low | Low | Some Concerns |

ROBINS-I (Risk of Bias in Non-randomized Studies-of Intervention) (NRSI - ROBINS-I)

| Domain | Bias due to confounding | Bias in selection of participants into the study | Bias in classification of interventions | Bias due to deviations from intended interventions | Bias due to missing data | Bias in measurement of outcome | Bias in selection of the reported result | Overall bias |
| --- | --- | --- | --- | --- | --- | --- | --- | --- |
| Naiki T, 2025 | Critical | Serious | Low | Moderate | Low | Moderate | Moderate | Critical |
| Hollis BW, 2013 | Serious | Serious | Low | Serious | Moderate | Moderate | Moderate | Serious |
| Ullevig SL, 2021 | Critical | Serious | Moderate | Serious | Moderate | Low | Moderate | Critical |
| Marshall DT, 2012 | Serious | Serious | Low | Moderate | Moderate | Moderate | Low | Serious |
| Van der Eecken H,2024 | Serious | Moderate | Low | Low | Moderate | Moderate | Moderate | Serious |
| deVere White RW, 2004 | Serious | Moderate | Low | Low | Moderate | Moderate | Moderate | Serious |

Cohort study (Newcastle-Ottawa Scale, NOS)

| Bias domain | Signalling question | Schenk JM, 2023 | Gregg JR, 2019 |
| --- | --- | --- | --- |
| Selection  (★★★★) | 1. Representativeness of the exposed cohort | ★ | ★ |
|  | 2. Selection of the non-exposed cohort | ★ | ★ |
|  | 3. Ascertainment of exposure |  | ★ |
|  | 4. Demonstration that outcome of interest was not present at start of study | ★ | ★ |
| Comparability  (★★) | 1. Comparability of cohorts on the basis of the design or analysis controlled for confounders | ★★ | ★★ |
| Outcome  (★★★) | 1. Assessment of outcome | ★ | ★ |
|  | 2. Was follow-up long enough for outcomes to occur? | ★ |  |
|  | 3. Adequacy of follow up of cohorts | ★ |  |
| Overall evaluation | | ★★★★★★★★ | ★★★★★★★ |

JBI Critical Appraisal Checklist for Analytical Cross Sectional Studies

| Evaluation item | Moreel X, 2014 | Moussa H, 2019 |
| --- | --- | --- |
| 1. Were the criteria for inclusion in the sample clearly defined? | Yes | Yes |
| 2. Were the study subjects and the setting described in detail? | Yes | Yes |
| 3. Was the exposure measured in a valid and reliable way? | Yes | Yes |
| 4. Were objective, standard criteria used for measurement of the condition? | Yes | Yes |
| 5. Were confounding factors identified? | Yes | Yes |
| 6. Were strategies to deal with confounding factors stated? | Yes | Yes |
| 7. Were the outcomes measured in a valid and reliable way? | Yes | Yes |
| 8. Was appropriate statistical analysis used? | Yes | Yes |
| Overall evaluation | High | High |
